# Self-assembly of keratin peptides: Its implication on the performance of electrospun PVA nanofibers

**DOI:** 10.1038/srep36558

**Published:** 2016-11-04

**Authors:** Kavitha Kadirvelu, Nishter Nishad Fathima

**Affiliations:** 1 Chemical laboratory, CSIR-CLRI, Adyar, Chennai -600020, Tamil Nadu, India

## Abstract

Drawing inspiration from the field of designer self-assembling materials, this work is aimed to focus on the self-assembling nature of extracted peptides. Hair keratin, a proteinacious reject in tanning industry has been chosen since they have been extracted and used for wide range of applications. Keratin source was subjected to five hydrolysis treatments (viz., sulphitolysis, β-mercaptoethanol, ionic liquid, thioglycolic acid and alkali) and assayed for functional groups. This was followed by the prediction of secondary structure using circular dichroism, determining the microstructural level to which the extracted peptide has self-assembled. Sulphitolysis and thioglycolic acid based hydrolysates exist in monomeric conformation, whereas β-mercaptoethanol based hydrolysate exhibited dimeric conformation. The subsequent part of the study is to incorporate these peptides into the nanofibers to study the structural implication of keratin peptides on its characteristics. Accordingly, the peptides were electrospun with PVA and subjected to morphological, mechanical, thermal and biological characterizations. Monomeric nanofiber mat has high tensile strength of around 5.5 MPa and offered lower mass transport resistance, whereas dimeric mat has high T_m_ of around 290 °C and was more biocompatible. These results help in understanding the extraction-structure-function aspect of the hydrolysates stressing the role of extraction methods on the choice of application.

The structure-property relationship of proteins/peptides has been explored in detail since time immemorial^1^. This concept has helped in designing peptides, specific for a particular application^2^. The strategy to incorporate these designed macromolecules into higher order structures, to serve high end applications is on the rise^3^. Drawing inspiration from such research activities, the influence of naturally occurring proteins extracted at different structural levels were studied on a nanofibrous scaffold. Keratin, a fibrous protein found in hair, feather, scales and hooves of animals has been used as the source of study^4^. Hair keratin, one of the α-helical proteinacious reject in the tanning industry has major impact on the pollution load caused by the industry^5^. This is due to the fact that hair is a complex biological composite present as a tight network of fibers embedded in a matrix and hence they are highly resistant to any degradation. So focus has been on the processing of hair and deriving a potential product from it^6^. Feed, fertilizers, cosmetics, coatings and composites are some of the common areas of application. With the advent of new technologies, the potential of keratin can be tapped for interesting applications.

To harvest the functional property of keratin it has to be obtained in a soluble form. This is possible by means of hydrolysis reactions. It is proved that the product obtained after hydrolysis behaves as true proteins and not as product of hydrolysis^4^. This extraction of keratin in solution form can be achieved through conventional methods like reduction/oxidation and other innovative approaches. Generally, these procedures can be classified under chemical, physicochemical and biological methods^7^. Oxidation or reduction methods^8^ are the most commonly employed chemical treatments. Physicochemical treatments involves steam explosion^9^, microwave based approach^10^ etc. Use of microbes or microbial enzymes to extract keratins comes under the third category^11^. These extraction techniques have effect on the protein structure and hence on the functional properties of keratin depending on the reaction conditions. In order to study the effect of this extraction-structure-property relationship, a sensitive and high potential structure like nanofiber mats becomes an obvious choice^12^. Nanofibers from keratin hydrolysates^13^ have already been reported and are found to be a high potential material for biological applications like drug delivery, tissue engineering, antimicrobial textiles and wound healing^14^.

These nanofibers possess several amazing characteristics such as very large surface area to volume ratio, flexibility in surface functionalities, and superior mechanical performance compared with any other known form of the material^12^. Particularly, electrospun nanofibers are in much demand compared to nanofibers fabricated by other techniques. This is due to the fact that, electrospinning technique can efficiently be scaled-up, can yield long continuous fibers, can exert control over the dimensions of fibers desired, can induce superior functionality and is reproducible compared to drawing, phase separation, template synthesis and self assembly based techniques^15^. It is a technique where the viscoelastic force of the polymer solution is overcome by the electrostatic force supplied, in order to yield polymeric fibers. Nanofibers fabricated from a protein/peptide-polymer solution have unique advantages in several disciplines^16^. Several works have already been reported, which explores the potential of this peptide-polymer hybrid in tailoring the characteristics of biomaterials. In a study by Stephens *et al*., the effect of electrospinning on the changes occurring in the secondary structure of the silk protein was studied using FT-Raman sepectroscopy^17^. Conversely, Rathna *et al*., describes the influence of protein-polymer solution together on the morphology of nanofibers^18^. Here, the viscosity and conductivity of the hybrid solution is concentrated upon to study the morphology of nanofibers fabricated. However, the influence of the structure of the peptide/protein on the nanofiber characteristics has not been addressed till date.

This study aims to fill this void and will offer strong foundation for designing nanofibrous biomaterials based on the structure of protein/peptide used. Apart from this it also gives us an insight on selection of the right choice of hydrolysate to suit application demands. Five different extraction procedures viz., sulphitolysis, β-mercaptoethanol, ionic liquid, thioglycolic acid and alkali based methods were carried out on the keratin source. Then the hydrolysates were characterized for their functional groups and secondary structure. The secondary structure was assessed by circular dichroism spectropolarimeter (CD) whereas the concentration of functional groups was determined by 2, 4, 6 - trinitrobenzene sulfonate (TNBS), 2, 2’-biquinoline-4, 4’-dicarboxylic acid/Bicinchoninic acid (BCA) and 5, 5’-dithiobis (2-nitrobenzoic acid) (DTNB) (Sigma)/Ellman’s assays. The hydrolysates were then used in combination with Poly (vinyl alcohol) (PVA) to fabricate electrospun nanofibers. PVA is widely used in biological applications since they are hydrophilic, biocompatible, biodegradable and highly flexible^19^. Though PVA/keratin nanofibers have already been reported, the combination was mainly chosen such that the effect of peptide on nanofiber characteristics can be concentrated upon rather than fabrication. The hybrid nanofibers were characterized for its morphological, thermal, mechanical and biological properties to correlate the influence of extracted peptides on fiber characteristics.

## Results and Discussion

### Characterization of keratin hydrolysates

#### Functional group analysis

**Figure 1:**
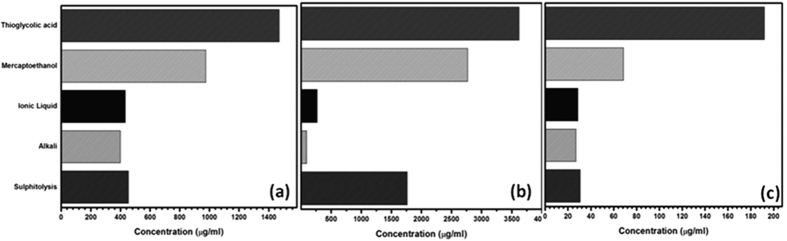
Bar plot of concentration of functional (**a**) Amine groups (**b**) Peptide bonds (**c**) Sulfhydryl groups of hydrolysates.

Functionality of a polymer depends on the number of functional groups available and hence quantitative determination of the reactive groups proved to be vital study in this work. Apart from the reactive nature, it also has an effect on the stability and conformational rigidity of the extracted protein^20^. The hydrolysates were characterized employing TNBS assay for primary amine groups, BCA assay for peptide bonds and DTNB/Ellman’s assay for sulfhydryl groups. It was found that the thioglycolic acid based hydrolysate possess increased concentration of functional groups followed by β-mercaptoethanol, sulphitolysis, ionic liquid based and alkali based hydrolysate (Fig. 1). The alkali based hydrolysates has set the minimum limit, which may be due to the harsh and unspecific interaction. In case of ionic liquids, the presence of scaly layer over the surface would have prevented the ionic liquid from interacting with the protein component. Hence, pre-treatment of the substrate is necessary to increase the extraction yield, which is a separate study in itself. The other three hydrolysates were found to have substantial level of functional groups, which is due to the combined action of reducing and denaturing agents used. Also the BCA assay can be used as an indicator for protein concentration, from which the extraction yield for the hydrolysis methods has been found out. It is found that thioglycolic acid based method yields more protein (36.2%) followed by β-mercaptoethanol (27.2%), sulphitolysis (17.63%), ionic liquid (2.7%) and alkali (1%) based hydrolysate.

#### Secondary structure analysis: Circular dichroic spectroscopic studies

**Figure 2:**
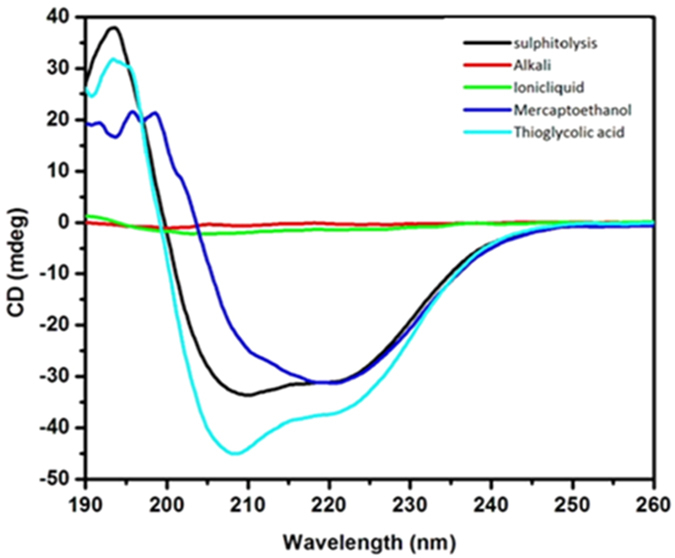
CD spectra of keratin hydrolysates after different hydrolysis.

The different types of hydrolysates obtained were analyzed for changes in their secondary structure using CD, since determination of existence of basic ordered structures gives an idea about the possible interaction of one subunit with another. α-helical CD spectrum of keratin is characterized by presence of two negative bands at 222 nm and 208 nm, and a positive band at 190 nm. This presence of three bands corresponding to the α-helical structure is as a result of n-π* and π-π* transitions. n-π* is responsible for the large negative band at 222 nm and because of exciton coupling π-π* transition was split into two resulting in a positive band at 190 nm and negative band at 208 nm^21^. The characteristic bands of hydrolysates (Fig. 2) were analyzed and were found that the alkali and ionic liquid based hydrolysates do not show any CD peak. This complements the results obtained through the assays as discussed in preceding sections. As stated earlier since there is decrease in yield for alkali and IL based hydrolysis the other three hydrolysates have been considered for further studies.

### Fabrication and characterization of keratin/PVA nanofiber mat

#### Fabrication

**Figure 3:**
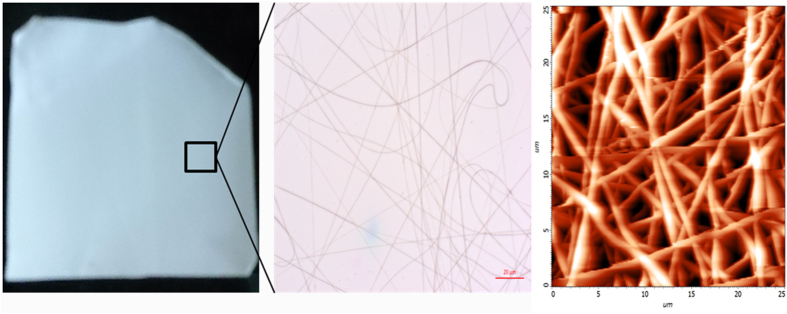
Optical and AFM micrographs of fabricated Keratin/PVA nanofibers (The average diameter was found to be 671.5 nm in case of optical micrograph and 638.2 nm in case of AFM micrograph).

In order to achieve nanofiber mat of keratin/PVA with beadless morphology and desired diameter (>500 nm), the various controllable parameters like flow rate, TCD (tip to collector distance), voltage applied, were simultaneously adjusted to arrive at a steady state electrospinning process. The effect of these parameters on the fiber characteristics have been studied extensively in several studies^12^. Based on the optical micrograph (Fig. 3), it was found that experiment, which was designed to obtain fibers at a flow rate of 0.4 ml/h, 18 kV voltage and 22 cm TCD yielded nanofibers of desired characteristics. The nanofiber mat was formed maintaining these conditions over a period of 3 h for all the three samples.

#### Characterization: Morphological

On observing the 3D pattern (Fig. 3) obtained through AFM analysis it is clear that the fibers have been randomly oriented and their average diameter is around 500–800 nm. It is also observed that the fibers are beadless thereby proving that forces involved in electrospinning are balanced enough to prevent breakup of polymer jet.

#### Characterization: Thermal

**Figure 4:**
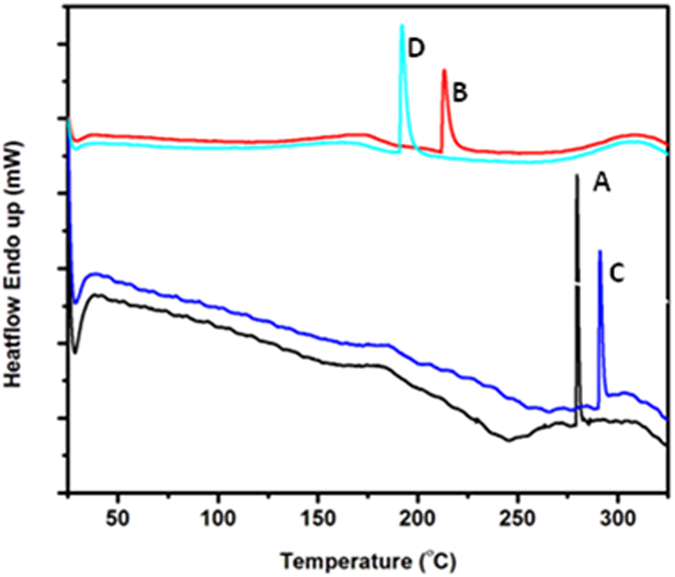
DSC thermograms of (**A**) Control (**B**) Sulphitolysis (**C**) β-Mercaptoethanol (**D**) Thioglycolic acid based keratin/PVA nanofibrous mats.

**Figure 5:**
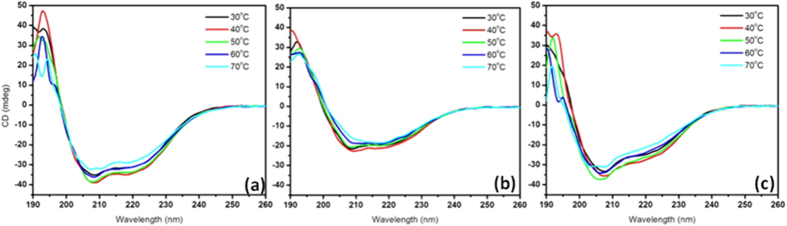
Temperature dependent CD spectra of keratin hydrolysates obtained by (**a**) Sulphitolysis (**b**) β-Mercaptoethanol (**c**) Thioglycolic acid based methods.

The melt thermograms (Fig. 4) has shown shift in the T_m_ values of keratin/PVA nanofibers as compared to that of PVA nanofibers, thus highlighting the effect of keratin on the property of nanofiber mat. It was found that there was a decrease in T_m_ of nanofibrous mat of all hydrolysates except for β-mercaptoethanol based. This decrease could be due to the fact that there is a decrease in the crystallinity of the polymer due to the interaction with protein subunits^18^. However, β-mercaptoethanol based mat showed a higher T_m_ value as compared to the control, in spite of the protein-polymer interaction. The logic behind this increase was assumed to be due to the structural differences. Hence conformational changes of the hydrolysates at an increasing temperature range were studied through circular dichroic spectroscopic analysis. Accordingly, spectra at a temperature interval of 5 °C were recorded from 30–75 °C (Fig. 5). The ratio of CD at 222 nm to that of CD at 208 nm^22^ gives the information about the adapted conformation of protein in that particular temperature. The CD_222_/CD_208_ values for the hydrolysates at a temperature range of 30–75 °C with 5 °C interval was calculated (Table 1). The change in CD_222_/CD_208_ ratio (due to helical content difference) causes variable interaction between adjacent peptides, which orients it in a particular way. This provides unique functional characteristics to these peptides thereby allowing them to respond variably to a particular stimulus (thermal treatment in our case). This is in correlation with molecular dynamic simulations of peptides with different CD_222_/CD_208_ ratio reported earlier^23^. Thermal stability studies revealed that the sulphitolysis based hydrolysate possessed increased helical content (CD_222_/CD_208_ value of 0.8). The ratio for thioglycolic acid based hydrolysates was found to be in the range of 0.7–0.6, thus proving the existence of lower helical content compared to that of sulphitolysis based hydrolysate. β-mercaptoethanol based hydrolysate was found to have CD_222_/CD_208_ value over 1, which corresponds to the existence of coiled-coil conformation. It is this difference over increasing temperature that has rendered the β-mercaptoethanol based mat thermostable. According to Greenfield *et al*., it is the hydrophobicity of the residues at the interface between 2 α-helices in a coiled-coil, which determines the thermal stability. The study claims that increased interface hydrophobicity resulted in an increase of 8 °C in T_m_^24^. The thermal stability characteristic could be explored indirectly towards the improvement of mechanical strength, thereby increasing the application potential of the mat.

#### Characterization: Porosity

**Figure 6:**
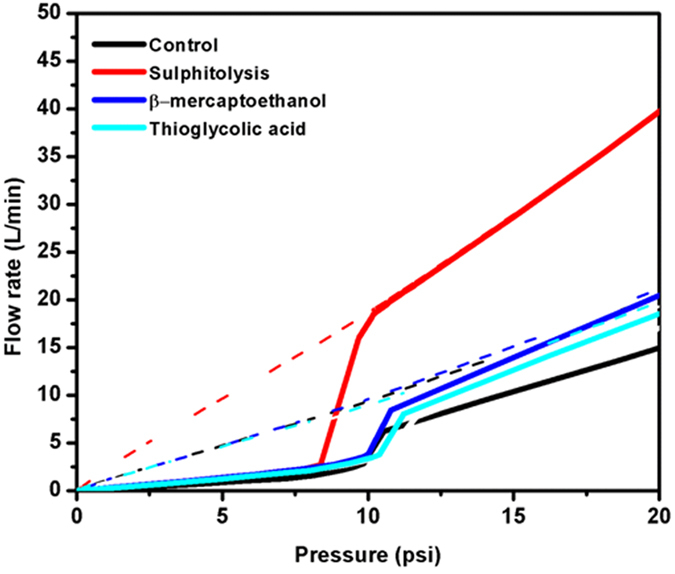
Wet and dry curves of the Keratin/PVA nanofibrous mat.

The pore characteristics of the obtained mat were studied through capillary flow porometry. A wetting liquid with low surface energy is used to fill the pores of the sample, followed by the increase in pressure of a non-reactive gas to empty the pore. This differential pressure required to displace the wetting liquid is related to pore diameter according to the following equation (1):


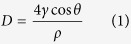


where D is the pore diameter, γ is surface tension of wetting liquid, θ is the contact angle of wetting liquid and p is differential pressure^25^. It is clear from the equation that larger pores require lower pressures to be emptied and samples with smaller pores require higher pressure. Thus, a careful choice of pressure needs to be chosen not only to sensitively measure the characteristics but also to prevent the pore structure distortion. Thus, measurement of differential pressure and flow rates through wet and dry samples gives an account of porosity characteristics of the fabricated nanofiber mats. The wet and dry curves obtained for keratin based nanofibrous mats along with PVA mat as control (Fig. 6) were analysed.

The trend of the plot is in such a way that in case of wet sample (thicker continuous line), initially there is no flow since the pores are occupied by the wetting liquid. At a specific pressure, the largest pore is emptied and the flow rate increases steadily, until all the pores are empty. Also the flow rate through the wet sample should match up to the dry sample (thin dotted line) flow rate at a specific pressure to offer low mass transport resistance^26^. Here, the trend of the curves was similar in all fibrous mats. However, it is the sulphitolysis based nanofibrous mat that showed significant increase in flow rate at a pressure of 6 psi, whereas the other mats nearly required a pressure around 10 psi to show the rise. Also the flow rate through the wet and dry samples is equalled at a pressure of 10 psi for sulphitolysis based mat, whereas the other mats did not show such a trend. Thus it is evident that sulphitolysis based mat possess better mass transport property compared to other mats. This could be a clear cut advantage, if the fabricated mats are to be used for any membrane based applications. The reason could be attributed due to the monomeric nature of keratin in the sulphitolysis based mat. According to the molecular dynamics (MD) simulation study of Yaakov Levy and Amedo Caflisch, the monomeric protein units are considered to be more flexible than the dimeric subunits. They have postulated that, the –N and –C termini are more flexible in case of monomeric unit whereas it is constrained in dimeric subunits^27^. Thus, the flexibility of the monomeric sulphitolysis based hydrolysate, has played a role in increasing the randomness of deposition, which has contributed to the increase in porosity. Thioglycolic acid based mat though possessing increased yield, due to lower helical content does not possess same intensity as that of sulphitolysis based mat. The higher porosity of mats obtained from sulphitolysis based hydrolysate, lowers the mass transport resistance thereby making them a favourable choice for membrane based applications.

#### Characterization: Mechanical

**Figure 7:**
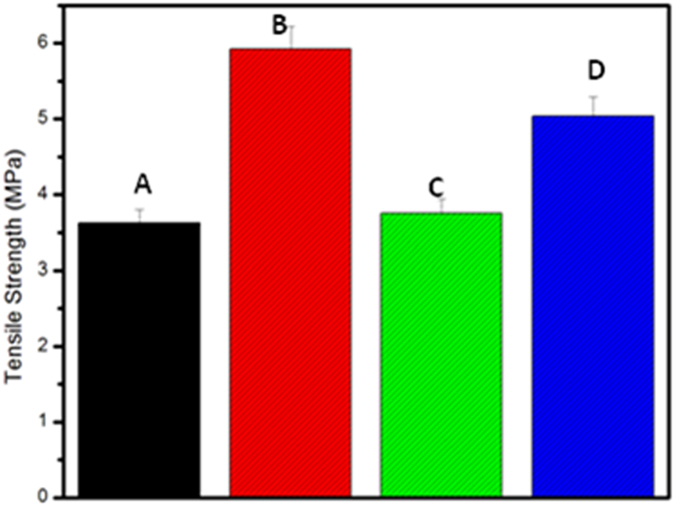
Bar plot of tensile strength values of (**A**) Control (**B**) Sulphitolysis (**C**) β-Mercaptoethanol (**D**) Thioglycolic acid based Keratin/PVA nanofibrous mats.

The tensile strength studies of the nanofibrous mat can be used as an index to measure the stiffness of the mat. The results obtained from the analysis (Fig. 7) shows that sulphitolysis based nanofibrous mat has high tensile strength of around 6 MPa followed by thioglycolic acid based mat with a strength of 5 MPa. The β- mercaptoethanol based mat was found to possess strength around 3–4 MPa. The superior mechanical stability of sulphitolysis based hydrolysate could be attributed, to the higher helical content of the hydrolysate. In a study reported by Kageshima *et al*., it is found that the hydrogen bonds, which are formed parallel to the direction of helix axis, are responsible for the longitudinal mechanical properties of the helix. Here it is postulated that when a α-helix is subjected to tensile force, the stretching of hydrogen bonds followed by its breakdown takes place. Then the backbone chain can be deformed without restraint, thereby decreasing the stiffness of the molecule. Only when the molecule reaches taut conformation by further stretching, the hydrogen bonds in the remaining part are stretched and broken, increasing the stiffness^28^. Thus the helical nature has prolonged the time to attain stiffness, which leads to the ripping apart of nanofibrous mat. The thioglycolic acid based mat has lower strength compared to sulphitolysis based mat, inspite of possessing higher yield due to the lower helical content in the extract.

#### Characterization: Biological

**Figure 8:**
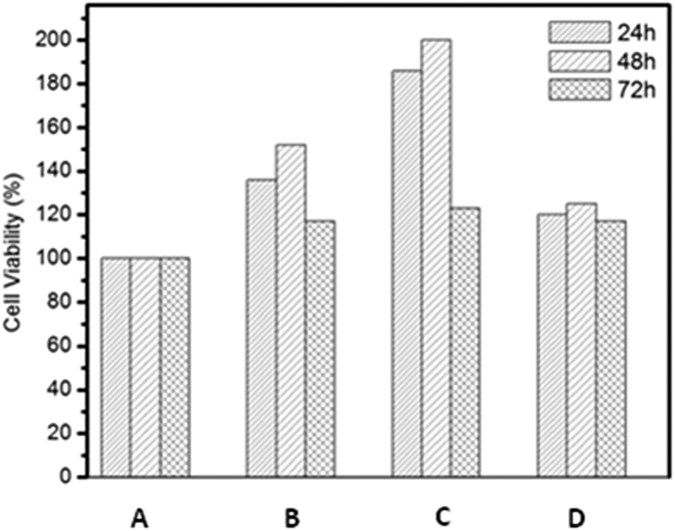
Bar plot of cell viability values of (**A**) Control (**B**) Sulphitolysis (**C**) β-Mercaptoethanol (**D**) Thioglycolic acid based Keratin/PVA nanofibrous mats at different time interval.

**Figure 9:**
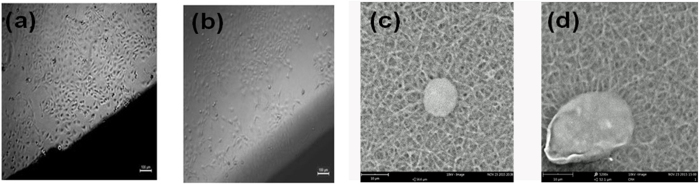
PCM image of (**a**) Dimeric mat (**b**) Monomeric mat (**c**) SEM micrograph of HaCat cell attached to control (PVA) mat (**d**) SEM micrograph of HaCat cell attached to Keratin/PVA nanofibrous mat.

The fabricated mats were evaluated for its cytocompatible characteristics using the MTT assay. Prior to this assay, the fabricated mats were thermally crosslinked^19^ at a temperature of 75 °C for a period of one week. The HaCat cell viability on different hydrolysate based nanofibrous mats for a period of 3 days was monitored (Fig. 8). The plot clearly shows increased percentage of cell viability on β- mercaptoethanol based hydrolysate containing nanofibrous mats than on others. This study clearly shows that there is no correlation in either helicity content or protein content affecting the viability, since both sulphitolysis (higher helicity) and thioglycolic acid (higher yield) based nanofibrous mat did not show increased viability percentage as that of β- mercaptoethanol based mat. On reviewing the literature it was found that coiled-coil motif of the protein aids in protein-protein recognition and association^29^. It is along the hydrophobic pocket of the dimer, that the binding of adhesion pathway mediated molecules takes place. Thus the dimeric nature (coiled-coil domain) of the β- mercaptoethanol based hydrolysate would have facilitiated contact between the cell adhesion molecules present on the surface of the cells and the nanofibrous matrix. Apart from improving the thermal characteristics, the coiled-coil nature of the hydrolysates have increased the cytocompatibility (skin cell compatibility in particular) of mat considerably, thus making them a favorable choice for biological applications. Also it was found that all samples have good cytocompatibity characteristics than the control due to the presence of functional sequences in the extracted peptides The PCM micrographs (Fig. 9) complement the above interpretation showing increased cell growth on β- mercaptoethanol based nanofibrous mat. SEM micrograph reveals that the cells have good affinity towards the mats. This significant finding will help in rationally designing high performance biomaterials which can be specifically engineered to regulate biological activity.

## Conclusion

In summary, the effect of extraction procedures on the peptides property to self-assemble followed by the peptide’s structural effect on nanofiber characteristics has been elucidated. The experimental conditions associated with each extraction method have created variable microenvironment for the peptides to self-assemble. This has imparted the peptide with inherent property, which was characterized and has been proposed for appropriate applications. While all the hydrolysate containing mats were characterized, it was only sulphitolysis and β-Mercaptoethanol based mat, which showed evident change correlating the structure-property relationship. Here the sulphitolysis based hydrolysate was found to have relatively the highest mechanical stability. The helical content has favored higher porosity of mats, lowering the mass transport resistance thereby making them a preferable choice for membrane applications. The coiled-coil β-Mercaptoethanol based hydrolysates apart from improving the thermal characteristics also increased the cytocompatibility of mat considerably, thus making them an obvious choice for biological applications. Though the structural effects of the other three hydrolysates did not have an impact on the nanofibers property their solution characteristics can be tapped for other applications. Thioglycolic acid based hydrolysates were found to be rich in reactive groups, which make them suitable for applications demanding presence of high functionality. The alkali based hydrolysate can be used in applications where neither the presence of functional groups nor the secondary structure has any role (as feed, fertilizer etc.). The green approach, Ionic liquid based hydrolysate has to be optimized further to increase the extraction yield to bring about an effect. This study thus clearly elucidates the role of extraction in affecting the property of the hydrolysates, thereby providing a direction in which proteinacious wastes can be efficiently utilized to generate functional materials. Also it paves a new direction in the field of electrospun materials where the structure of the protein or peptide can be looked upon before generating a functional material for a specific application.

## Materials and Methods

### Selection of keratin source

Enzymatically removed goat hair, obtained from tannery division of CSIR-CLRI (Chennai, India) was utilized as the keratin source. The hair obtained was cleaned thoroughly and soaked overnight in 1:1 ratio of chloroform and methanol, to remove stains, grease or any fatty residues. If % nitrogen content in the source is 13.5 or above, the material can be considered relatively pure protein and can be hydrolyzed without further treatment^30^. In keratin since the nitrogen content is around 16%^31^ the hair was cleaned without requiring special treatment. The cleaned fibers were then snipped and subjected to various hydrolysis treatments.

### Preparation of keratin hydrolysates

1 wt% of cleaned and snipped keratin fibers was taken and subjected to sulphitolysis^32^, alkali^33^, 1-butyl-3-methyllimidazolium chloride (BMIM^+^ Cl^−^) (Aldrich, India) ionic liquid^34^, β-mercaptoethanol^35^ (Aldrich, India) and thioglycolic acid^36^ (Aldrich, India) based treatments according to previously reported procedures.

In brief, in case of sulphitolysis, the hair fibers were treated with 8 M urea, 0.5 M sodium metabisulfite and 0.05 M SDS. The pH was adjusted to 6.5 using 5N NaOH. Then the solution was kept under stirring at 65 °C for 3 hours and was filtered. In the alkali based method, the hair fibers were treated with 0.5N NaOH at 75 °C for 3 hours and then filtered. The ionic liquid based method involved treatment of hair fibers with 10% solution of BMIM^+^ Cl^−^ in a controlled temperature setup under constant mechanical stirring and in an inert atmosphere (N_2_). Here the temperature maintained was 120 °C for a time period of 24 hours. The hair fibers were subjected to constant stirring along with urea, SDS, β-mercaptoethanol for over 12 hours at 60 °C for β-mercaptoethanol based method. Thioglycolic acid based preparation involved treating the hair fibers with 0.2 M thioglycolic acid adjusted to pH 9.6 with 3 M KOH at 30 °C for 3 hours. Then 8 M urea was added to the reaction vessel and reaction continued for 24 hours at the same temperature. Then the solution was adjusted to pH 7 by drop wise addition of acetic acid. Following this, the solution was stirred for 3 days to effect oxidation which can be monitored by the progressive disappearance of mercapto specific odor. The hydrolysis procedure was carried out as described except for the amendment that instead of wool, goat’s hair was used as the keratin source.

Hydrolysates obtained through above procedures were filtered through a 110 mm Ø Whatman filter paper. Then the filtrates were dialyzed against distilled water in cellulose tubing (1.3 inch, M.W cut-off 14,000) (Sigma-Aldrich, India) for 3 days with intermittent water changes. The dialysate was then centrifuged to remove insoluble precipitates if any and the subsequent water soluble keratin was used for further studies.

### Characterization of keratin hydrolysates

The concentration of primary amine groups was determined through TNBS (5% w/v in H_2_O, Sigma) assay^37^, which was used as an indicator for determining the degree of hydrolysis. To estimate the concentration of protein, BCA (Sigma Aldrich) assay^38^ was carried out. The presence of sulfhydryl groups, like that of amine groups has serious implications on the reactive nature of the protein. In this study, DTNB (Sigma)/Ellman’s assay^39^ was performed to analyze the sulfhydryl group content.

The secondary structure of the protein samples were estimated using Jasco J-815 CD spectrometer. The instrument was operated at a nitrogen atmosphere level of 5 LPM in the standard sensitivity range. The samples were diluted in the ratio of 1:3 with distilled water and were loaded in a quartz cuvette of 0.1 cm path length. The scan was performed in the wavelength range of 190–260 nm. Thermal stability of the samples was analyzed in the temperature range of 30–75 °C with 5 °C interval.

### Fabrication of keratin/PVA nanofibers using electrospinning

8% PVA (M.W 140,000, HiMedia) was dissolved in respective hydrolysates as such. The peptide-polymer solution was set to overnight stirring to obtain homogeneity, without application of heat. They were then subjected to eletrospinning using “Espin Nano Model V1 setup”. Aluminium plate was used as collector to allow for the deposition of randomly aligned nanofibers. The electrospinning conditions employed include, applied voltage of 18 kV, flow rate of 0.4 ml/h and tip to collector distance of 22 cm. These conditions were carefully chosen after thorough optimization to yield beadless nanofibers.

### Characterization of keratin/PVA nanofibers

The Novex B series microscope was used for preliminary studies to analyze the optimum range of parameters that yield beadless fibers of desired dimensions. NT-MDT’s NTEGRA aura AFM was employed to checkout for the diameter range and morphology of the obtained nanofiber mats. Semi-contact mode was employed for scanning the surface of the mat using silicon nitride tip.

Porous materials Inc’s advanced automated humid air porometer HCFP-1100 AE was used in analyzing the pore characteristics of nanofibers. Capillary flow porometry is the mode of measurement of pore properties.

Netzsch’s DSC-204 F1 Phoenix^®^Differential Scanning Calorimeter was used to obtain thermograms of different keratin hydrolysate/PVA nanofiber mats. The temperature range studied was from 25 to 350 °C. Here 350 °C was chosen as the upper limit, since PVA as such possess melting point of 230 °C. This extended limit is to account in for the increase in stability (if any).

Instron 3345 universal tensile tester machine was used to study the mechanical properties of the nanofiber mat according to the ASTM standard of D882-12 which is a test method for measuring tensile properties of thin plastic sheeting. The mats were punched into strips of 10 × 70 × 0.1 mm^3^ at three different sites and were subjected to tensile stress at a cross head speed of 5 mm min^−1^.

The cytocompatibility (skin cell) of the nanofiber mat was assessed by MTT (3-(4,5-dimethylthiazol-2-yl)-2,5-diphenyltetrazolium bromide salt) (Sigma, India) assay. The mat was snipped into small pieces of uniform dimension, placed in 24-well tissue culture plates and sterilized under UV radiation. HaCaT (immortalized human keratinocytes) cells of 20,000 cells/ml concentration were seeded and grown for 3 days at 37 °C in a 5% CO_2_ and 95% O_2_ humidified incubator. Samples were harvested on 1^st^, 2^nd^ and 3^rd^ day for analysis. MTT reagent was added at a concentration of 500 μL/well and incubated for 4 hours. Subsequently, 200 μL DMSO was added to solubilise the formed formazan crystal by metabolically active cells. Absorbance was measured at 570 nm using BioRad ELISA plate reader. The assay was carried out in triplicates.

## Additional Information

**How to cite this article**: Kadirvelu, K. and Fathima, N. N. Self-assembly of keratin peptides: Its implication on the performance of electrospun PVA nanofibers. *Sci. Rep.* **6**, 36558; doi: 10.1038/srep36558 (2016).

**Publisher’s note:** Springer Nature remains neutral with regard to jurisdictional claims in published maps and institutional affiliations.

## Figures and Tables

**Table 1:** Temperature based helical propensity identification of keratin hydrolysates via determining CD_222_/CD_208_ ratio.

Temperature (°C)	CD_222_/CD_208_ Sulphitolysis based hydrolysate	CD_222_/CD_208_ β - Mercaptoethanol based hydrolysate	CD_222_/CD_208_ Thioglycolic acid based hydrolysate
**30**	0.8608	0.8728	0.7101
**35**	0.8344	0.8830	0.7313
**40**	0.8486	0.8840	0.7415
**45**	0.8323	0.9066	0.7022
**50**	0.8411	0.8763	0.6928
**55**	0.8282	0.9061	0.6820
**60**	0.8337	0.9513	0.6662
**65**	0.8348	0.9762	0.6625
**70**	0.8492	1.0721	0.6450
**75**	0.8599	1.1440	0.6560
**Existence**	**α – Helical** (**high content**)	**Coiled- Coil**	**α – Helical** (**low content**)
